# A novel assay based on DNA melting temperature for multiplexed identification of SARS-CoV-2 and influenza A/B viruses

**DOI:** 10.3389/fmicb.2023.1249085

**Published:** 2023-12-19

**Authors:** Peng Gao, Yanyan Fan, Xiaomu Kong, Rui Zhang, Lida Chen, Yongwei Jiang, Yi Liu, Meimei Zhao, Guoxiong Deng, Yongtong Cao, Liang Ma

**Affiliations:** ^1^Department of Clinical Laboratory, China-Japan Friendship Hospital, Beijing, China; ^2^Laboratory of Clinical Microbiology and Infectious Diseases, Department of Pulmonary and Critical Care Medicine, China-Japan Friendship Hospital, Beijing, China; ^3^National Center for Clinical Laboratories, Beijing Hospital, National Center of Gerontology, Beijing, China; ^4^Department of Blood Transfusion, China-Japan Friendship Hospital, Beijing, China

**Keywords:** SARS-CoV-2, influenza viruses, simultaneous detection, differentiation, melting curve analysis

## Abstract

**Introduction:**

The severe acute respiratory syndrome coronavirus-2 (SARS-CoV-2) and influenza viruses can cause respiratory illnesses with similar clinical symptoms, making their differential diagnoses challenging. Additionally, in critically ill SARS-CoV-2–infected patients, co-infections with other respiratory pathogens can lead to severe cytokine storm and serious complications. Therefore, a method for simultaneous detection of SARS-CoV-2 and influenza A and B viruses will be clinically beneficial.

**Methods:**

We designed an assay to detect five gene targets simultaneously via asymmetric PCR-mediated melting curve analysis in a single tube. We used specific probes that hybridize to corresponding single-stranded amplicons at low temperature and dissociate at high temperature, creating different detection peaks representing the targets. The entire reaction was conducted in a closed tube, which minimizes the risk of contamination. The limit of detection, specificity, precision, and accuracy were determined.

**Results:**

The assay exhibited a limit of detection of <20 copies/μL for SARS-CoV-2 and influenza A and <30 copies/μL for influenza B, with high reliability as demonstrated by a coefficient of variation for melting temperature of <1.16% across three virus concentrations. The performance of our developed assay and the pre-determined assay showed excellent agreement for clinical samples, with kappa coefficients ranging from 0.98 (for influenza A) to 1.00 (for SARS-CoV-2 and influenza B). No false-positive, and no cross-reactivity was observed with six common non-influenza respiratory viruses.

**Conclusion:**

The newly developed assay offers a straightforward, cost-effective and nucleic acid contamination-free approach for simultaneous detection of the SARS-CoV-2, influenza A, and influenza B viruses. The method offers high analytical sensitivity, reliability, specificity, and accuracy. Its use will streamline testing for co-infections, increase testing throughput, and improve laboratory efficacy.

## 1 Introduction

Infection with severe acute respiratory syndrome coronavirus-2 (SARS-CoV-2) causes coronavirus disease 2019 (COVID-19), which has become a sustained global pandemic (Lai et al., 2020; Sharma et al., 2020). As of May 4, 2023, the COVID-19 pandemic has affected nearly every country, with more than 752 million cases and 6.9 million deaths reported worldwide (World Health Organization, 2023). Individuals infected with SARS-CoV-2 can exhibit a range of clinical symptoms, from an asymptomatic course to severe acute respiratory distress syndrome, with most individuals showing mild symptoms or remaining asymptomatic (Gao et al., 2021). Although symptomatic individuals are a common source of transmission, undiagnosed asymptomatic cases can transmit the virus, thus increasing the prevalence of COVID-19 infection (Gao et al., 2021). The clinical symptoms of SARS-CoV-2 are very similar to those caused by the influenza A and B viruses, including fever, cough, dyspnea and fatigue (Mayuramart et al., 2021; Havasi et al., 2022). However, the prevention and treatment strategies for different viral infections are distinct. Several clinical studies have reported that co-infection with SARS-CoV-2 and one or more influenza viruses or other pathogens, particularly among severe COVID-19 cases, can exacerbate patients’ condition (Nowak et al., 2020; Wu et al., 2020; Huang et al., 2021). Thus, rapid detection and accurate identification of SARS-CoV-2 and other influenza viruses are critical for providing precise treatment, controlling the infection source and implementing effective prevention measures. Accordingly, an accurate and convenient assay that can simultaneously detect SARS-CoV-2 and influenza A and B viruses will be of great clinical benefit.

The feasibility of a detection system to be applied in clinical laboratories is influenced by various factors, including instrument cost, laboratory space, complexity, testing capability, handling time, and result time (Burd, 2010). An efficient detection method offers enhanced detection capability and reduces the burden on the detection system, which helps free up staff resources and reduce turnaround times. Generally, virus isolation based on cell culture is the “gold standard” for diagnosing viral diseases, but it is time-consuming and complex (Chen et al., 2020). At present, numerous nucleic acid-based molecular detection methods have been developed for the simultaneous detection of SARS-CoV-2 and influenza viruses. Reverse transcription-polymerase chain reaction (RT-PCR) is a widely used method that provides high sensitivity, specificity, and good repeatability (Jokela et al., 2010; Tang et al., 2020; Zhang et al., 2020). To date, several commercial detection kits based on RT-PCR have been developed and used, such as the CDC’s Influenza SARS-CoV-2 (Flu SC2) Multiplex Assay (Shu et al., 2021), the Roche Cobas^®^ SARS-CoV-2 and Influenza A/B Assay (Kosai et al., 2022), and Cepheid’s Xpert Xpress SARS-CoV-2/Flu/RSV (Leung et al., 2021). The use of multiple detection methods based on RT-PCR mainly depends on two detection modes. The first mode is uses different fluorophore-labeled probes that target various genes, and the results are determined according to the colors of fluorescent probes. However, this mode has the disadvantage of crosstalk between fluorescence spectra, which can reduce the accuracy of the results, and this method requires labeling with fluorescent dye probes of different colors and reacting in detection instruments with multiple fluorescent channels, which are associated with higher costs. The second mode involves dividing a sample into several reaction units in which different target genes will be amplified independently. However, this strategy requires a greater amount of viral RNA input and increases the workload of laboratory staff, thus reducing the efficiency of detection.

Except for RT-PCR, reverse-transcription loop-mediated isothermal amplification (RT-LAMP) is another molecular technique to diagnose COVID-19 that grabbed the attention of many scientists due to its simplicity and high specificity (Aoki et al., 2021; de Oliveira Coelho et al., 2021). It is a rapid, and cost-effective technique that diagnoses viral diseases by targeting and amplifying six regions in the cDNA, using 4–6 primers (Notomi et al., 2015). However, the high number of primers increases the possibility of forming primer dimers, leading to false-positive results. So, the main limitation lies in primer design, a time-consuming and complex process requiring high expertise (Alhamid et al., 2022). Droplet digital PCR (ddPCR) was previously developed to reduce false-negatives results limitation reported in RT-PCR because it can accurately detect the virus in samples with low viral load (Hindson et al., 2011). Nevertheless, ddPCR requires expensive instrumentations and is time-consuming (Falzone et al., 2020; Behera et al., 2021). Clustered regularly interspaced short palindromic repeat (CRISPR)-Cas system has been developed to detect influenza or SARS-CoV-2 infections (Broughton et al., 2020). CRISPR-based technology offers advantages due to its simplicity, speed, and cost-effectiveness. It can provide results within 30 min up to 1 h and does not require expensive instruments (Broughton et al., 2020; Aman et al., 2021). However, the possible “off-target” phenomenon can affect the judgment of the test results. The non-specific collateral cleavage of Cas12 and Cas13 systems may influence other target pathogens, which can make it challenging to develop multiplex detection (Zhang et al., 2022). Whole-genome sequencing methods have been utilized to determine the origin of SARS-CoV-2 (Ren et al., 2020) and monitor the emergence of mutations and new variants (Harilal et al., 2020; John et al., 2021). However, it requires complex equipment and highly trained personnel. The high cost and long assay time make it unsuitable for large-scale testing. Moreover, sequencing errors may occur due to a large number of reads or low viral loads in clinical samples (Slatko et al., 2018). Therefore, a new method with high accuracy, efficiency and cost-effectiveness is needed for simultaneous detection of SARS-CoV-2 and influenza A/B viruses.

Multiplex asymmetric PCR based detection has evolved a rapid and convenient tool in wide variety of bioanalyses (Zhu et al., 2007; Yu et al., 2022; Kong et al., 2023). It uses many pairs of unequal concentrations of the primers for amplification, generating single stranded amplicons. This facilitates the simultaneous amplification of many targets of interest in one reaction, thus enhancing assay throughput and allowing more efficient use of each sample. Previous study has reported the development of multiplex polymorphism detection in a single tube for screening folate metabolism genes (Yu et al., 2022). Additionally, it has been utilized to identify seven immune-escape RBD mutations of Omicron (Kong et al., 2023), as well as detecting drug resistance genes in Enterobacteriaceae (Zhu et al., 2007). In the current study, we aimed to employ multiplex reverse transcription (RT) asymmetric PCR combined with melting curve analysis (MCA) to simultaneously detect SARS-CoV-2, influenza A/B viruses in a single reaction, which could be applied in poorly equipped primary hospitals and laboratories, as well as in scenarios demanding urgent diagnoses.

## 2 Materials and methods

### 2.1 Primer and probe design

The one-pot RT-asymmetric PCR-combined MCA assay was designed to target the ORF1ab gene and nucleocapsid (N) gene for the detection of SARS-CoV-2. Detection of influenza A and B was achieved by targeting the matrix (M) and hemagglutinin (HA) genes, respectively. The human RPP30 gene was selected as an internal reference and can serve as an indicator of the specimen quality. Primer and probe sequences targeted highly conserved regions of the influenza A, B and SARS-CoV-2 genome and were based on the published literature (van Elden et al., 2001; Duchamp et al., 2010; Dunn et al., 2014; Wei et al., 2020). The SARS-CoV-2 ORF1ab gene and N gene probe, with a carboxy-X-rhodamine (ROX) reporter/QSY quencher, and probes for the influenza A M gene, influenza B HA gene, and human RPP30 gene probe, with a carboxyfluorescein (FAM) reporter/QSY quencher, were purchased from Sangon (Sangon Biotech, Shanghai, China). Five pairs of primers and probes were used to simultaneously detect the three viruses in a single-tube. The optimal sequences of primers and probes are listed in Table 1.

**TABLE 1:** Primers and probes for the detection of SARS-CoV-2, influenza A, influenza B in the RT- asymmetric PCR-combined MCA assay.

Target	Oligo	Primer/Probe sequence (5′-3′)	Length (nt)	Amplicon size (bp)
Influenza A M gene	Fwd	CTTCTAACCGAGGTCGAAACGTA	23	155
	Rev	GGTGACAGGATTGGTCTTGTCTTTA	25	
	Probe	5′-FAM- TACGTTCTCTCTATCATTCCATCA -BHQ1-3′	24	
Influenza B HA gene	Fwd	AAATACGGTGGATTAAACAAAAGCAA	26	170
	Rev	CCAGCAATAGCTCCGAAGAAA	21	
	Probe	5′- FAM-CACCCATATTGGGCAATTTCCTATGGC-BHQ1 -3′	27	
SARS-CoV-2 ORF1ab gene	Fwd	CCCTGTGGGTTTTACACTTAA	21	119
	Rev	ACGATTGTGCATCAGCTGA	19	
	Probe	5′- ROX-TCTGCGGTATGTGGAAAGGTT-BHQ2-3′	21	
SARS-CoV-2 N gene	Fwd	GGGGAACTTCTCCTGCTAGAAT	22	99
	Rev	CAGACATTTTGCTCTCAAGCTG	22	
	Probe	5′-ROX- TTGCTGCTGCTTGAC-BHQ2-3′	15	
Human RPP30 gene	Fwd	AAGGTATACAATTTCCAGTGCCC	23	109
	Rev	GTCATATGGCCCTCTTATTTCTAA	24	
	Probe	5′- FAM-ATGTAATTATATCTAGTGCT -BHQ1-3′	20	

### 2.2 Simulated single-stranded DNA (ssDNA) synthesis and viral RNA extraction

Simulated ssDNAs were designed to simulate the single-stranded amplicons that are generated through asymmetric PCR and were used to simulate the MCA process of the single-stranded amplicon and probe and to evaluate the melting temperature (Tm) value of the detection peak. The simulated ssDNAs were purchased from Sangon (Sangon Biotech) and are listed in Supplementary Table 1.

RNA templates were extracted from MS2-based virus-like particles (MS2-VLPs) corresponding to different viruses as well as the collected clinical samples. The specific virus sequences packaged in MS2-VLPs were quantified by digital PCR, and the results were regarded as the required positive standards. Viral RNA was extracted and purified with the Gene Rotex96 nucleic acid extraction and purification system (Tianlong Science and Technology Co., Ltd., Xi’an, China) using an RNA extraction kit (Tianlong Science and Technology Co., Ltd.) in accordance with the manufacturer’s instructions. The concentration and purity of the extracted RNA samples were assessed using a NanoDrop™ 2000 spectrophotometer (Thermo Fisher Scientific, Waltham, MA, United States). The qualified RNA samples were subsequently frozen and stored at −80°C.

### 2.3 One-pot RT-asymmetric PCR-combined MCA assay

The assay was performed on the SLAN-96P real-time PCR system (Shanghai Hongshi Medical Technology Co., Ltd). The Abstart One-step RT-PCR Mix (Sangon Biotech, Shanghai, China) was used to reverse transcription and PCR amplification. Each 50-μL PCR mixture contained 2 μL Abstart Taq^®^ Polymerase with dNTP (0.125 U/μL) supplied in 5 × One-step RT-PCR Buffer (with 3.3 mM Mg^2+^), 5 × Solution I buffer, with 0.01–0.2 μM limiting primers, 0.08–1.6 μM excess primers, 0.005–0.2 μM probes, and 15 μL RNA template. The concentration details for the primers and probes are presented in Supplementary Table 2. The reactions were incubated at 42°C for 30 min and 95°C for 5 min followed by 50 cycles of 94°C for 30 s, 60°C for 60 s, and 72°C for 30 s, and then incubated at 72°C for 10 min, 95°C for 2 min, and 30°C for 2 min. The reaction mixture was subjected to MCA from 30 to 90°C and finally incubated at 40°C for 20 s. To avoid contamination of the PCR products, the whole reaction is performed in a closed PCR amplification tube that is never opened during assay performance.

### 2.4 Limits of detection (LoDs)

MS2-VLPs have been widely used as quality control materials for detecting pathogenic RNA viruses (Sun et al., 2013; Wang et al., 2015a; Zhang et al., 2016) due to their similarity in structure to natural viruses, stability, RNase resistance, non-infectivity and inability to replicate by itself both *in vivo* and *in vitro* (Sun et al., 2013; Wang et al., 2015a,b). Previous studies showed that MS2-VLPs were used to evaluate the LOD and precision of 7 commercial real-time PCR kits for Zaire ebolavirus (Wang et al., 2015b). Recent studies showed that SARS-CoV-2-MS2-VLPs was used to assess the LOD of rRT-PCR assays when detecting SARS-CoV-2 variants (Chen et al., 2022), as well as be used to evaluate the external quality assessment for molecular detection of SARS-CoV-2 in clinical laboratories (Wang et al., 2021). In our study, the analytical sensitivity was determined quantitatively using MS2-VLPs that contained the SARS-CoV-2 ORF1ab gene and N gene, the influenza A M gene, and the influenza B HA gene, separately. For each type of MS2-VLPs, the concentration was calibrated by digital PCR to 1.0 × 10^3^ copies/μL. The three types of MS2-VLPs were mixed equally and serially diluted 10-fold with TE buffer to 500, 250, 125, 62.5, 31.3, 15.6, and 7.8 copies/μL. Twenty replicates of each dilution were tested and the lower LoD was defined as the concentration in copies/μL of the lowest dilution that could be detected with 95% probability and determined by probit analysis.

### 2.5 Specificity

The specificity of each primer and probe oligonucleotide sequence was evaluated by conducting a BLAST analysis^1^ using the sequences of SARS-CoV-2, influenza A, and influenza B against the nr/nt database of the National Center for Biotechnology Information and the Global Initiative on Sharing All Influenza Database^2^ to ensure that the primers and probes accurately matched the target gene sequence. Supplementary Figure 1 showed supporting information to better present the blastn analysis.

To determine the specificity of the developed assay, a species-specific sample panel was created that included clinical samples containing other respiratory viruses from patients who had clinical symptoms overlapping with those of influenza infection and COVID-19 (*n* = 58). The clinical samples were obtained from the Department of Respiratory and Critical Care Medicine at China-Japan Friendship Hospital (Beijing, China) and this study was approved by the Institutional Review Board of the China-Japan Friendship Hospital (No. 2022-KY-248), China. The sample panel contained human rhinovirus (*n* = 25), human parainfluenza virus (*n* = 12), adenovirus (*n* = 4), respiratory syncytial virus (*n* = 6), and human bocavirus (*n* = 1), all of which were positively detected via their respective RT-PCR assays. Approximately, 1 ng/μL of each viral RNA (15 μL) was used in the assay.

### 2.6 Precision

The precision of the developed assay was assessed in terms of both intra-assay precision (repeatability) and inter-assay precision (reproducibility). The variability of the developed assay was evaluated by detecting three concentrations (500, 125, and 31.3 copies/μL) of equally mixed positive MS2-VLPs. To observe intra-assay variability, each concentration was analyzed five times in one reaction. To observe inter-assay variability, each concentration was analyzed five times in independent reactions performed by different users on separate days. The variability of the Tm value was evaluated using variable analysis.

### 2.7 Accuracy

To evaluate the accuracy of the developed assay, a total of 345 clinical samples were collected and tested, including samples containing influenza A, influenza B, SARS-CoV-2, and co-infection viruses. The pre-determined testing for SARS-CoV-2 was conducted by using the Novel Coronavirus (2019-nCoV) Nucleic Acid Detection Kit [BioGerm Medical Technology Co., Ltd. (Shanghai, China)], while the influenza viruses were using the Influenza A Virus and Influenza B Virus Detection Kit [Coyote Bioscience Co., Ltd. (Beijing, China)]. The clinical samples were obtained from the Department of Respiratory and Critical Care Medicine at China-Japan Friendship Hospital (Beijing, China) and this study was approved by the Institutional Review Board of the China-Japan Friendship Hospital (No. 2022-KY-248). The positive clinical samples selected for this study were identified through pre-determined testing, with Ct values spanning the range of positivity (as indicated in Table 2). Detailed Ct values information for the positive samples were listed in Supplementary Table 3. For influenza A, a total of 111 samples were collected, including 40 positives and 71 negatives, and the specimen types included nasopharyngeal swabs (*n* = 73), oropharyngeal swabs (*n* = 38). For influenza B, 105 previously tested samples (29 positives and 76 negatives) were tested, and the specimen types included nasopharyngeal swabs (*n* = 78) and oropharyngeal swabs (*n* = 27). For SARS-CoV-2, a total of 129 previously diagnosed samples (42 positives and 87 negatives) were tested, including nasopharyngeal swabs (*n* = 102), and oropharyngeal swabs (*n* = 27). In addition, two samples with co-infection of influenza A and SARS-CoV-2 and one sample with co-infection of influenza B and SARS-CoV-2 were tested to assess the ability of the assay to detect co-infection, and the specimen types included nasopharyngeal swab (*n* = 1) and oropharyngeal swabs (*n* = 2). The detailed clinical samples information was presented in Supplementary Table 4.

**TABLE 2:** The Ct value distribution of selected positive samples.

	SARS-CoV-2			Influenza A	Influenza B
	ORF1ab gene	N gene		M gene	HA gene
Ct range*			Ct range^#^		
≤20.0	10	8	≤20.0	3	3
20.1–25.0	11	11	20.1–25.0	15	14
25.1–30.0	11	12	25.1–30.0	12	9
>30	10	11	>30	10	3
Total positive	42		Total positive	40	29
Total Negative	87		Total Negative	71	76

*Ct values were obtained by routine SARS-CoV-2 testing using the Novel Coronavirus (2019-nCoV) Nucleic Acid Detection Kit [BioGerm Medical Technology Co., Ltd. (Shanghai, China)]; ^#^Ct values were calculated by adding 15 cycles to the raw Ct values obtained by routine Influenza viruses testing using the Influenza A Virus and Influenza B Virus Detection Kit [Coyote Bioscience Co., Ltd. (Beijing, China)], as this assay detects fluorescence signals from the 16th PCR cycle. Ct, cycle threshold; SARS-CoV-2, severe acute respiratory syndrome coronavirus-2.

### 2.8 Statistical analysis

Basic statistical values, including mean, standard deviation, and coefficient of variation for the mean Tm (°C) value, were calculated using Excel (Microsoft Corp., Redmond, WA, USA). The LoD for each virus was calculated using probit regression analysis, which determines the concentration at which the target is successfully detected in 95% of replicates (Miller et al., 2019). Overall percent agreement, positive percent agreement (PPA), negative percent agreement (NPA), and Cohen’s kappa coefficient with associated 95% confidence intervals (95% CI) were calculated using VassarStats online.^3^ Cohen’s kappa values were interpreted according to Landis and Koch (1977).

## 3 Results

### 3.1 Principle of the one-pot RT- asymmetric PCR-combined MCA assay

**FIGURE 1:**
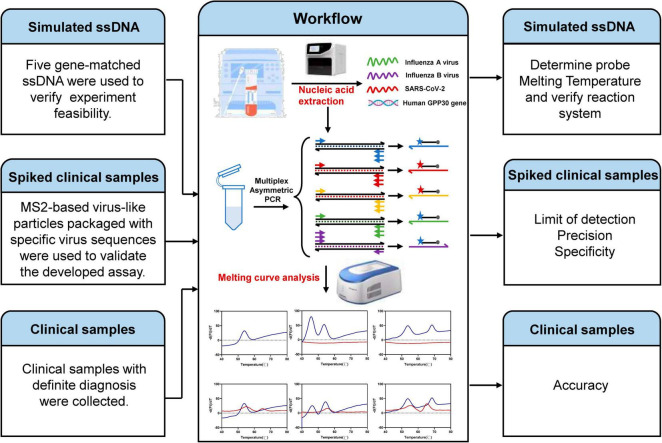
Schematic illustration of one-pot RT-asymmetric PCR-combined MCA assay.

**FIGURE 2:**
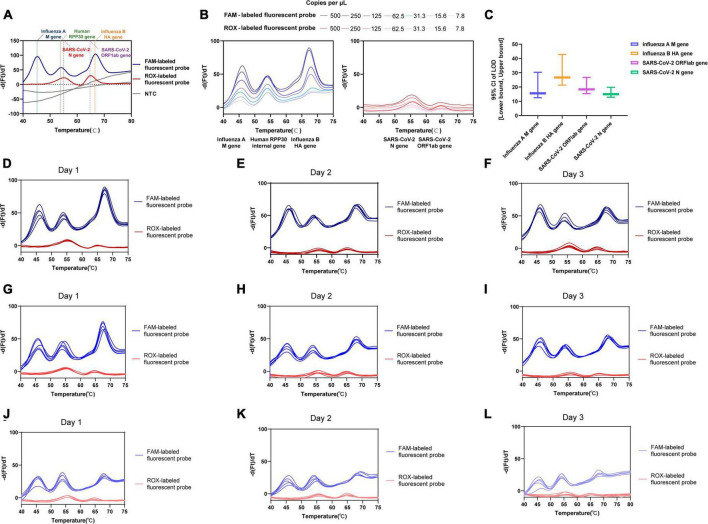
Validation performance for one-pot RT-asymmetric PCR-combined MCA assay. **(A)** The feasibility analysis confirmed by using simulated ssDNAs. **(B)** The determined LoD for each target using dilutions of SARS-CoV-2, influenza A and B virus MS2-VLPs. **(C)** The LoD [95% CI: (Lower bound, upper bound)] of the assay for the virus targeted genes. **(D–L)** The intra-assay precision and inter-assay precision assessed by three concentrations (500, 125, and 31.3 copies/μL) of equally mixed positive MS2-VLPs. **(D–F)** 500 copies/μL of equally mixed positive MS2-VLPs was analyzed five times in independent reactions performed by different users on three days, respectively. **(G–I)** 125 copies/μL of equally mixed positive MS2-VLPs was analyzed five times in independent reactions performed by different users on three days, respectively. **(J–L)** 31.3 copies/μL of equally mixed positive MS2-VLPs was analyzed five times in independent reactions performed by different users on three days, respectively.

We developed a rapid and cost-efficient method for simultaneous detection of the SARS-CoV-2 and influenza A/B viruses. The RNA templates were extracted from both MS2-VLPs and clinical samples and then added into the prefabricated reaction solution for one-pot detection. As shown in Figure 1, the assay consisted of two steps. First, the extracted viral RNA was initially reverse transcribed into cDNA. Due to the insufficient amount of the generated cDNA, we then used cDNA as the template to amplify and generate enough amount of single stranded amplicons through asymmetric PCR. The asymmetric PCR uses unequal concentrations of the primers for amplification. In the early stage of amplification, both primers are available the double-stranded amplicons can be generated exponentially through normal PCR. When the limiting primer is depleted in the reaction mixture, only the still available excess primer is able to continue the amplification of target cDNA and thereby produces single stranded amplicons. Therefore, we generated sufficient amount of distinct detectable viral single-stranded amplicons in one reaction by increasing different viral pairs of primers. Subsequently, the designed fluorescent-labeled probes specifically bind to the corresponding viral single-stranded amplicons to produce detectable peaks by MCA. The feasibility of the method was verified using simulated ssDNAs, and the assay results can be directly visualized as a specific single peak by MCA (shown in Figure 2A). This assay can detect five targeted genes using probes labeled with two different fluorophores, allowing for the simultaneous detection of three viruses, which has many advantages, including short amplification time, low cost, easy accessibility, and high-throughput detection capability.

### 3.2 Turnaround time

Considering the differences in operational time caused by various sample preparation and RNA extraction methods, our evaluated turnaround time merely refers to the RNA detection time. In a single experiment, it is feasible to simultaneously detect 96 samples, yielding results in roughly 3 h. Since only the extracted viral RNA needs to be added, the hands-on time is reduced, which in turn reduces exposure risk, particularly given the specificity of COVID-19.

### 3.3 LoDs

The LoD for each target was determined using probit analysis with dilutions of SARS-CoV-2 and influenza A and B virus MS2-VLPs (Table 3 and Figure 2B). The calculated LoD values were 18.37 copies/μL (95% confidence interval [CI]: 15.395–26.758 copies/μL) for the SARS-CoV-2 ORF1ab gene, 14.96 copies/μL (95% CI: 12.862–19.805 copies/μL) for the SARS-CoV-2 N gene, 15.60 copies/μL (95% CI: 12.516–30.316 copies/μL) for the influenza A M gene, and 26.70 copies/μL (95% CI: 21.304–42.783 copies/μL) for the influenza B HA gene. The SARS-CoV-2 N gene showed the lowest LoD and was the most sensitive among the four targets. Additionally, the analytical sensitivity was slightly and nominally lower for influenza B than for influenza A and SARS-CoV-2 (Figure 2C). These results indicate that the assay has high sensitivity, which supports its evaluation as a potential diagnostic tool.

**TABLE 3:** Results of LOD for multiplex detection based on RT- asymmetric PCR-combined MCA assay.

Targeted gene	No. of replicates detected at each dilution/total no. of replicates at indicated no. of copies per μL							
	**1000**	**500**	**250**	**125**	**62.5**	**31.3**	**15.6**	**7.8**
Influenza A M gene	20/20 (100%)	20/20 (100%)	20/20 (100%)	20/20 (100%)	20/20 (100%)	20/20 (100%)	19/20 (95%)	11/20 (55%)
Influenza B HA gene	20/20 (100%)	20/20 (100%)	20/20 (100%)	20/20 (100%)	20/20 (100%)	20/20 (100%)	11/20 (55%)	6/20 (30%)
SARS-CoV-2 ORF1ab gene	20/20 (100%)	20/20 (100%)	20/20 (100%)	20/20 (100%)	20/20 (100%)	20/20 (100%)	17/20 (85%)	5/20 (25%)
SARS-CoV-2 N gene	20/20 (100%)	20/20 (100%)	20/20 (100%)	20/20 (100%)	20/20 (100%)	20/20 (100%)	20/20 (100%)	3/20 (15%)

SARS-CoV-2, severe acute respiratory syndrome coronavirus-2.

### 3.4 Specificity

The specificity of the developed assay was verified in two ways. First, silico analysis and blastn analysis were performed, and the results showed that the primers and probes could match the target gene sequences correctly, with no evidence of non-target matches observed. Second, the assay’s specificity was evaluated by measuring cross-reactivity against 58 human respiratory clinical samples known to contain several different diagnostic respiratory viruses. Table 4 showed that the assay returned negative results for the other respiratory viruses and the blank control, with no amplification of the corresponding nucleic acids. These results showed that a specificity of 100% was achieved, with no cross-reactivity with any of the viruses tested.

**TABLE 4:** Results of respiratory viruses included in the specificity assessment.

Clinical samples of other respiratory viruses	No. tested	Identified results from diagnostic assays	Results from this study		
			Target: SARS-CoV-2	Target: influenza A	Target: influenza B
Adenovirus	4	Positive	Negative	Negative	Negative
Parainfluenza viruses	12	Positive	Negative	Negative	Negative
Respiratory syncytial virus	6	Positive	Negative	Negative	Negative
Rhinovirus	25	Positive	Negative	Negative	Negative
Human metapneumovirus	10	Positive	Negative	Negative	Negative
Human bocavirus	1	Positive	Negative	Negative	Negative

SARS-CoV-2, severe acute respiratory syndrome coronavirus-2.

### 3.5 Precision

All four targeted viral genes and the internal control gene were detected in the tested samples. We conducted a quantitative analysis of the coefficient of variation of the Tm value for each target by calculating the mean and standard deviation Tm values. The percent coefficient of variation (%CV) was calculated as a parameter representing intra-assay variability. Testing mixtures included 500 copies/μL (Figures 2D–F), 125 copies/μL (Figures 2G–I), and 31.3 copies/μL (Figures 2J–L) viral concentrations of the influenza A M gene, influenza B HA gene, and SARS-CoV-2 ORF1ab and N gene. The obtained intra-assay %CV values ranged from 0.12 to 1.16%. The inter-assay variability among three runs ranged from 0.34 to 0.99% for all targets, indicating that the assay achieved reliable detection across different viral loads. Supplementary Table 5 showed the detailed results of intra-assay precision and inter-assay precision.

### 3.6 Assay performance with clinical samples

**FIGURE 3:**
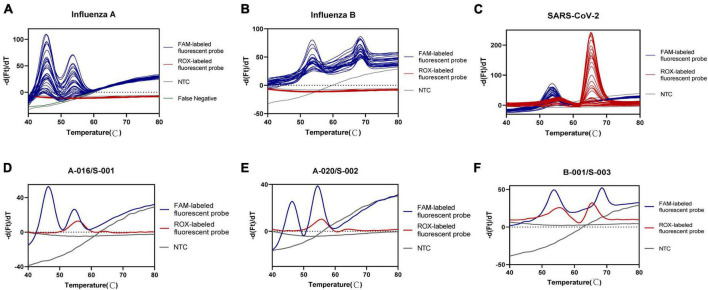
Accuracy analysis of one-pot RT-asymmetric PCR-combined MCA assay. **(A–C)** Detection peak of Influenza A, Influenza B, and SARS-CoV-2 positive clinical samples, respectively. **(D, E)** Detection peak of co-infection of Influenza A and SARS-CoV-2 positive clinical samples. **(F)** Detection peak of co-infection of Influenza B and SARS-CoV-2 positive clinical samples.

To assess the accuracy of the developed multiplex assay, a total of 345 clinical samples were tested. The overall percent agreement, as well as positive and negative percent agreement for influenza A were estimated at 99.1% (95% CI: 94.4–99.9%), 97.5% (95% CI: 85.3–99.9%), and 100% (95% CI: 93.6–100%), respectively. Only one specimen showed discordant results between our developed assay and the pre-determined assays. This specimen exhibited a weakly positive result for influenza A in the pre-determined assay (with Ct value of Influenza A gene:34.676, Ct value of internal gene:34.191 and Ct value less than 35 regarded as positive), whereas our assay result was negative. This discrepancy may be attributed to the very low presence of viral RNA in the oropharyngeal swab sample and potential RNA degradation. For SARS-CoV-2 and influenza B, our developed assay yielded results identical to the pre-determined assay. Cohen’s kappa statistic ranged from 0.98 (influenza A) to 1.00 (SARS-CoV-2 and influenza B), indicating nearly perfect agreement between the two assays. Detailed concordance metrics between two assays were shown in Table 5. The results for clinical positive samples were presented in Figures 3A–C. Moreover, this method accurately detected multiple viruses within samples, including cases of co-infection with two different viruses, such as two cases of co-infection with influenza A and SARS-CoV-2 (Figures 3D, E) and one case with influenza B and SARS-CoV-2 (Figure 3F). Additionally, we also observed no false positive results among the 234 samples that were predetermined to be negative for SARS-CoV-2, influenza A, and influenza B.

**TABLE 5:** Comparison of the performance between our developed assay and the pre-determined assay.

Pre-determined assay* (target)		Our developed assay			Overall percent agreement	Positive percent agreement	Negative percent agreement	Cohen’s kappa statistic
		Positive	Negative	Total	% (95% CI)	% (95% CI)	% (95% CI)	κ (95% CI)
SARS-CoV-2	Positive	42	0	42	100% (96.4–100%)	100% (89.6–100%)	100% (94.7–100%)	1 (1.00–1.00)
	Negative	0	87	87				
	Total	42	87	129				
Influenza A	Positive	39	1	40	99.1% (94.4–99.9%)	97.5% (85.3–99.9%)	100% (93.6–100%)	0.98 (0.94–1.00)
	Negative	0	71	71				
	Total	39	72	111				
Influenza B	Positive	29	0	29	100% (95.6–100%)	100% (85.4–99.7%)	100% (94.0–100%)	1 (1.00–1.00)
	Negative	0	76	76				
	Total	29	76	105				

*Pre-determined assay for routine SARS-CoV-2 testing were using the Novel Coronavirus (2019-nCoV) Nucleic Acid Detection Kit [BioGerm Medical Technology Co., Ltd. (Shanghai, China)]; Pre-determined assay for routine Influenza viruses testing were using the Influenza A Virus and Influenza B Virus Detection Kit [Coyote Bioscience Co., Ltd. (Beijing, China)]. PPA, positive percent agreement; NPA, negative percent agreement; SARS-CoV-2, severe acute respiratory syndrome coronavirus-2.

## 4 Discussion

From 2019 to 2023, SARS-CoV-2 has caused a global pandemic resulting in millions of deaths. Similar to other respiratory viruses, the influenza viruses cause annual epidemics, which also result in millions of infections and deaths worldwide. The symptoms of influenza vary from mild to severe, including extra-pulmonary complications such as viral myocarditis and encephalitis. Patients infected with SARS-CoV-2 or influenza viruses demonstrate similar clinical symptoms, which complicates the differential diagnosis of COVID-19 from respiratory illnesses caused by influenza viruses (Ma et al., 2020). Nucleic acid tests are necessary for a definitive diagnosis of infection with these viruses, but sequential testing for different pathogens can delay the application of mitigation strategies. In critically ill COVID-19 patients, co-infections with other respiratory pathogens pose a concern, as recent studies suggest they can lead to serious complications such as acute respiratory distress syndrome, fulminant myocarditis, acute kidney injury, and multiple organ failure (Kim et al., 2020; Ma et al., 2020). However, antiviral agents that target influenza A and B viruses can be effective at lowering patients’ risks of developing pneumonia, requiring hospitalization, and dying while hospitalized, making the simultaneous detection of SARS-CoV-2 and influenza A/B viruses as co-infecting pathogens critically important for informing the clinical management of patients.

The multiplex real-time reverse transcription PCR is the most widely used method for simultaneous detection of influenza A, influenza B and SARS-CoV-2 (Chung et al., 2021; Ni et al., 2021; Norz et al., 2021; Pabbaraju et al., 2021). This approach relies on probes labeled with different fluorophores to identify various virus gene targets. However, due to the requirement for distinct labeled fluorescein to emit fluorescence under specific excitation wavelengths, this method needs to be tested on the multi-channel fluorescence PCR instrument, which is associated with higher hardware costs. Considering the crosstalk factor between fluorescence spectra, there should be no more than four types of fluorescein-labeled probes in a single reaction (Xu et al., 2021). Therefore, when multiple targets are detected, the samples may be divided into several reaction units in which different target genes are amplified and detected independently (Mancini et al., 2021). This may also lead to an increase in the number of reagents and labor costs. In comparison with this method, our developed assay uses two types of fluorescein-labeled probes, which can be implemented on the dual-channel fluorescence PCR instrument, reducing device costs. Additionally, it can simultaneously detect five gene targets in a single reaction, thus reducing reagent and labor costs.

In the present study, our established assay was based on asymmetric PCR-mediated melting curve method in a one-pot detection system with an uncomplicated experimental procedure. The extracted viral RNA was first reverse transcribed into cDNA as template, and different pairs of viral primers in unequal concentrations were used to amplify and generate sufficient amount of different viral single-stranded amplicons through asymmetric PCR. Then, specific probes hybridize to their corresponding single-stranded amplicons at low temperature and dissociate as the temperature is increased during the MCA, resulting in different detection peaks representing the corresponding targets. In comparison to other fluorescent PCR methods, this approach utilizes two types of fluorophore-labeled probes and can be performed in a two-channel fluorescent PCR system, reducing detection costs. Moreover, we demonstrated that the method can detect five different gene targets in a single tube, substantially enhancing the sample detection throughput. The assay was validated and demonstrated to be highly accurate, sensitive and reliable. The LoDs for SARS-CoV-2 and influenza A were <50 copies per reaction, and that for influenza B was <80 copies per reaction, which is equivalent to LoD for the current commercial RT-PCR kit. No cross-reactivity was observed between gene targets, even at high viral loads, and no cross-reactivity was observed with six other common non-influenza respiratory viruses. The intra-assay and inter-assay precision with three viral concentrations for four targeted genes ranged from 0.12 to 1.16%, suggesting similar reliability with the current RT-PCR method. The assay demonstrated remarkable consistency with the pre-determined testing, accurately detecting positive samples covering different viral load levels. In addition, the entire reaction is conducted in a closed-tube, reducing the risk of contamination. Overall, the developed assay is simple, low cost, nucleic acid contamination-free, and can simultaneously detect and differentiate five gene targets in a two-channel fluorescent PCR system, which will streamline testing for coinfections, increase testing throughput, and improve the laboratory turnaround time.

Due to the strict management regulations on clinical samples of SARS-CoV-2 during the pandemic period, the number of clinical samples were limited, especially the co-infection clinical samples, which is a limitation of our study. Besides, this method incorporates a melting curve analysis, which entails an extra step compared to the traditional RT-PCR approach, thereby rendering it slightly more time-consuming. Therefore, we will further optimize the cycling conditions for reverse transcription and multiple asymmetric PCR amplification to achieve more advantage in terms of detection time. In addition, the broad temperature interval between detection peaks is another limitation, which resulted in only three gene targets being detected using the same fluorophore-labeled probe. To overcome this limitation, we plan to refine the probe and PCR reaction conditions, with the goal of reducing the temperature interval between each detection peak. We also aim to use fewer fluorescently labeled probes to detect more gene targets in the future.

In conclusion, the method presented in this study offers several benefits for the simultaneous detection of SARS-CoV-2 and influenza A/B viruses. These include: (a) comparable analytical sensitivity (with a LoD < 50 copies per reaction for SARS-CoV-2 and influenza A, and <80 copies per reaction for influenza B); (b) satisfactory reliability (with a coefficient of variation of Tm values between intra-assay and inter-assay precision for three virus concentrations of <1.16%); (c) high consistent performance in clinical specimen validation experiments; (d) prevention of PCR product contamination (as the entire PCR reaction is conducted in a closed tube); and (e) simple operation, cost-effectiveness and high testing throughput (with the ability to detect and differentiate three respiratory viruses and the human internal control target in a single reaction using two fluorophore-labeled probes, and the ability to conduct high-throughput sample testing in a 96- or 384-well PCR analyzer). Therefore, this study presents a significant and clinically validated assay that can be implemented for the detection of SARS-CoV-2 and influenza A/B viruses. We anticipate that this assay will prove beneficial during upcoming influenza seasons when influenza may co-circulate with SARS-CoV-2, as rapid detection of co-infections can provide valuable time for local health authorities to contain transmission and enable clinicians to provide appropriate treatments.

## Data availability statement

The original contributions presented in the study are included in the article/Supplementary material, further inquiries can be directed to the corresponding authors.

## Ethics statement

The studies involving humans were approved by the Institutional Review Board of the China-Japan Friendship Hospital. The studies were conducted in accordance with the local legislation and institutional requirements. The participants provided their written informed consent to participate in this study.

## Author contributions

All authors listed have made a substantial, direct, and intellectual contribution to the work, and approved it for publication.

## Footnotes

1
http://blast.ncbi.nlm.nih.gov/Blast.cgi

2
https://www.gisaid.org

3
http://vassarstats.net/index.html

## Supplementary material

Supplementary Figure 1 The supporting information presents the blastn analysis.
