# Supplementary figures and images for: A novel assay based on DNA melting temperature for multiplexed identification of SARS-CoV-2 and influenza A/B viruses
Source: Front Microbiol. 2023 Dec 19;14:1249085. doi: 10.3389/fmicb.2023.1249085 (PMC10762780; doi:10.3389/fmicb.2023.1249085)

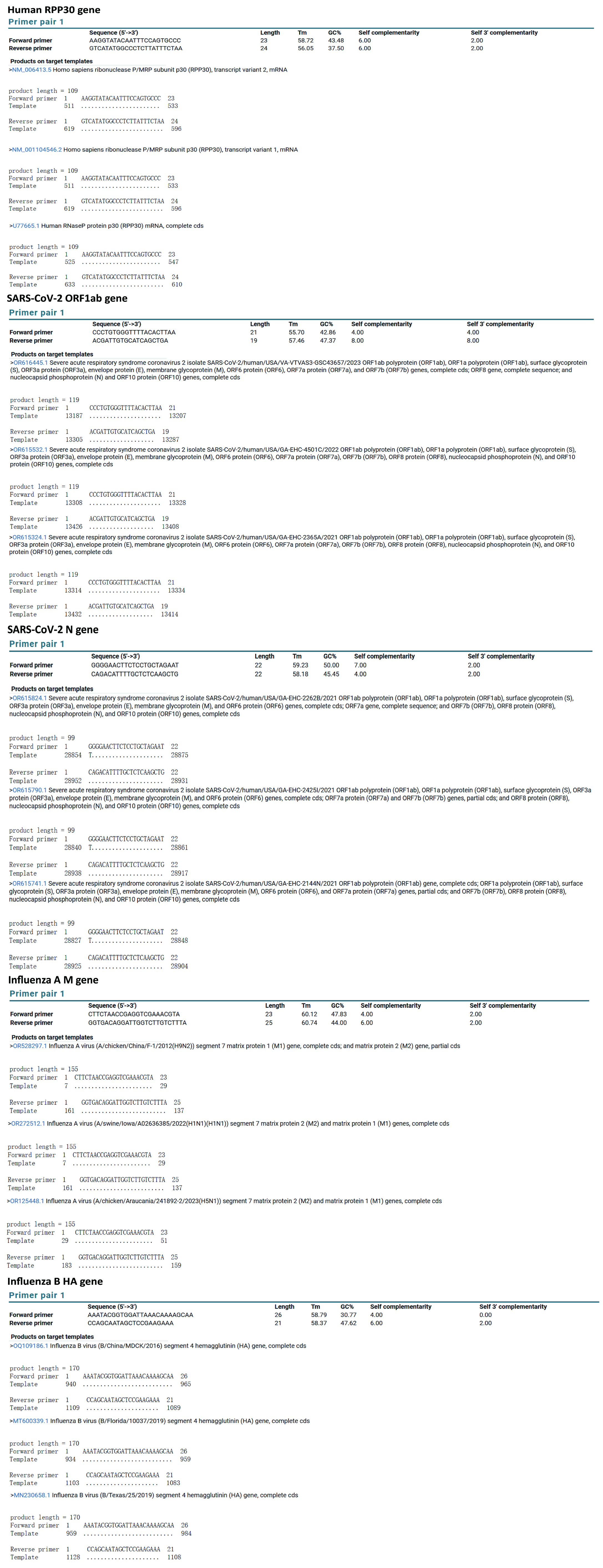

Supplement: Supplementary Figure 1 — The supporting information presents the blastn analysis. [file Image_1.tif]
