# Supplementary material for: A novel assay based on DNA melting temperature for multiplexed identification of SARS-CoV-2 and influenza A/B viruses
Source: Front Microbiol. 2023 Dec 19;14:1249085. doi: 10.3389/fmicb.2023.1249085 (PMC10762780; doi:10.3389/fmicb.2023.1249085)
Supplement: Supplementary file 2 [file Table_1.docx]

**Table S1. The synthesis sequences of the simulated ssDNAs**

| **Simulated ssDNA** | **Sequence (5’- 3’)** | **Length (nt)** |
| --- | --- | --- |
| SARS-CoV-2 ORF1ab gene | TACAGCCATAACCTTTCCACATACCGCAGACGGTACA | 37 |
| SARS-CoV-2 N gene | GTTCAATCTGTCAAGCAGCAGCAAAGCA | 28 |
| Influenza A M gene | GGGCCTGATGGAATGATAGAGAGAACGTACGTTT | 34 |
| Influenza B HA gene | CAAAAGCCATAGGAAATTGCCCAATATGGGTGAAAAC | 37 |
| Human RPP30 internal gene | TCTGCAGCACTAGATATAATTACATTCTTT | 30 |

**Table S2. Reaction mix composition for RT- asymmetric PCR-combined MCA assay.**

| **Component** | **Final Concentration** |
| --- | --- |
| Abstart Taq with dNTP | 2 μL |
| 5×One-step RT-PCR buffer | 1× |
| 10×Solution I | 1× |
| SARS-CoV-2 *ORF1ab* gene forward primer | 0.2 μM |
| SARS-CoV-2 *ORF1ab* gene reverse primer | 1.6 μM |
| SARS-CoV-2 *ORF1ab*-ROX probe | 0.2 μM |
| SARS-CoV-2 *N* gene forward primer | 0.01 μM |
| SARS-CoV-2 *N* gene reverse primer | 0.08 μM |
| SARS-CoV-2 *N*-ROX probe | 0.005 μM |
| Influenza A *M* gene forward primer | 0.1 μM |
| Influenza A *M* gene reverse primer | 0.8 μM |
| Influenza A *M*-FAM probe | 0.1 μM |
| Influenza B *HA* gene forward primer | 0.6uM |
| Influenza B *HA* gene reverse primer | 0.06uM |
| Influenza B *HA*-FAM probe | 0.06uM |
| Human *RPP30* gene forward primer | 0.1 μM |
| Human *RPP30* gene reverse primer | 0.8 μM |
| Human *RPP30*-FAM probe | 0.05 μM |

**Table S3. Detailed Ct value information of the clinical positive samples**

| **^*^Ct values for SARS-CoV-2 positive specimens** | | | | **^#^Ct values for Influenza A positive specimens** | | | **^#^Ct values for Influenza B positive specimens** | | |
| --- | --- | --- | --- | --- | --- | --- | --- | --- | --- |
| **Sample No.** | **RNP** | **ORFlab** | **N** | **Sample No.** | **Influenza A gene** | **Internal Control gene** | **Sample No.** | **Influenza B gene** | **Internal Control gene** |
| **S-001** | 22.277 | 33.574 | 34.285 | **A-001** | 19.286 | 31.926 | **B-001** | 30.756 | 31.871 |
| **S-002** | 30.371 | 31.254 | 32.090 | **A-002** | 31.895 | 30.980 | **B-002** | 24.371 | 29.629 |
| **S-003** | 29.512 | 25.684 | 25.605 | **A-003** | 27.262 | 30.785 | **B-003** | 29.504 | 31.855 |
| **S-004** | 26.980 | 29.176 | 29.520 | **A-004** | 24.363 | 30.105 | **B-004** | 19.221 | 27.855 |
| **S-005** | 31.707 | 31.973 | 33.598 | **A-005** | 27.316 | 31.926 | **B-005** | 18.092 | 29.512 |
| **S-006** | 37.316 | 18.504 | 18.980 | **A-006** | 29.848 | 30.395 | **B-006** | 31.512 | 28.551 |
| **S-007** | 28.293 | 27.996 | 29.074 | **A-007** | 20.887 | 28.871 | **B-007** | 19.976 | 29.590 |
| **S-008** | 26.941 | 24.277 | 24.559 | **A-008** | 28.488 | 29.215 | **B-008** | 28.262 | 31.691 |
| **S-009** | 27.918 | 18.621 | 20.059 | **A-009** | 24.082 | 29.848 | **B-009** | 27.504 | 29.168 |
| **S-010** | 26.738 | 23.324 | 23.918 | **A-010** | 31.918 | 31.355 | **B-010** | 25.160 | 28.605 |
| **S-011** | 26.301 | 15.918 | 16.434 | **A-011** | 30.293 | 32.387 | **B-011** | 26.465 | 25.582 |
| **S-012** | 26.746 | 26.863 | 26.949 | **A-012** | 24.160 | 30.324 | **B-012** | 24.121 | 29.207 |
| **S-013** | 27.770 | 27.559 | 26.762 | **A-013** | 21.418 | 28.207 | **B-013** | 30.223 | 30.137 |
| **S-014** | 27.988 | 14.754 | 16.215 | **A-014** | 20.816 | 31.074 | **B-014** | 27.652 | 30.879 |
| **S-015** | 25.973 | 20.645 | 21.715 | **A-015** | 23.887 | 31.012 | **B-015** | 23.855 | 29.613 |
| **S-016** | 25.871 | 18.449 | 19.316 | **A-016** | 24.098 | 29.113 | **B-016** | 22.988 | 28.918 |
| **S-017** | 24.246 | 28.613 | 29.348 | **A-017** | 32.207 | 32.629 | **B-017** | 23.395 | 30.105 |
| **S-018** | 22.254 | 15.926 | 17.207 | **A-018** | 29.191 | 31.098 | **B-018** | 24.082 | 28.012 |
| **S-019** | 25.762 | 20.207 | 20.863 | **A-019** | 34.676 | 34.191 | **B-019** | 28.543 | 31.449 |
| **S-020** | 24.348 | 16.402 | 16.848 | **A-020** | 25.301 | 32.293 | **B-020** | 27.582 | 30.223 |
| **S-021** | 23.801 | 24.770 | 25.621 | **A-021** | 30.676 | 29.738 | **B-021** | 22.918 | 28.543 |
| **S-022** | 28.316 | 17.676 | 19.715 | **A-022** | 27.309 | 29.941 | **B-022** | 24.809 | 29.215 |
| **S-023** | 27.246 | 16.098 | 17.059 | **A-023** | 19.237 | 31.051 | **B-023** | 22.238 | 29.848 |
| **S-024** | 29.176 | 23.090 | 24.324 | **A-024** | 21.527 | 30.059 | **B-024** | 24.848 | 28.941 |
| **S-025** | 26.918 | 21.129 | 22.277 | **A-025** | 29.637 | 28.855 | **B-025** | 23.254 | 29.059 |
| **S-026** | 29.176 | 23.090 | 24.324 | **A-026** | 19.285 | 29.605 | **B-026** | 24.910 | 30.973 |
| **S-027** | 26.113 | 22.996 | 24.473 | **A-027** | 26.723 | 29.809 | **B-027** | 22.129 | 29.410 |
| **S-028** | 26.309 | 24.426 | 25.230 | **A-028** | 24.262 | 29.887 | **B-028** | 23.980 | 30.035 |
| **S-029** | 26.324 | 19.957 | 21.184 | **A-029** | 20.996 | 30.348 | **B-029** | 25.121 | 29.441 |
| **S-030** | 29.996 | 28.051 | 29.074 | **A-030** | 26.895 | 30.895 |  |  |  |
| **S-031** | 27.091 | 26.059 | 26.816 | **A-031** | 29.410 | 31.629 |  |  |  |
| **S-032** | 26.082 | 30.527 | 31.387 | **A-032** | 20.730 | 30.262 |  |  |  |
| **S-033** | 29.770 | 27.324 | 28.785 | **A-033** | 29.191 | 29.738 |  |  |  |
| **S-034** | 25.137 | 33.082 | 33.543 | **A-034** | 30.168 | 31.191 |  |  |  |
| **S-035** | 28.480 | 28.949 | 30.301 | **A-035** | 21.340 | 32.066 |  |  |  |
| **S-036** | 25.863 | 34.332 | 34.559 | **A-036** | 21.168 | 29.168 |  |  |  |
| **S-037** | 29.848 | 32.949 | 33.730 | **A-037** | 31.285 | 31.020 |  |  |  |
| **S-038** | 23.246 | 26.504 | 27.520 | **A-038** | 30.832 | 30.535 |  |  |  |
| **S-039** | 27.762 | 28.652 | 29.066 | **A-039** | 24.395 | 30.949 |  |  |  |
| **S-040** | 28.582 | 30.285 | 30.223 | **A-040** | 31.762 | 30.425 |  |  |  |
| **S-041** | 28.004 | 31.160 | 32.457 |  |  |  |  |  |  |
| **S-042** | 27.605 | 31.926 | 32.301 |  |  |  |  |  |  |

^*^Ct values were obtained by routine SARS-CoV-2 testing using the Novel Coronavirus (2019-nCoV) Nucleic Acid Detection Kit (BioGerm Medical Technology Co., Ltd. (Shanghai, China); ^#^Ct values were calculated by adding 15 cycles to the raw Ct values obtained by routine Influenza viruses testing using the Influenza A Virus and Influenza B Virus Detection Kit Coyote Bioscience Co., Ltd. (Beijing, China), as this assay detects fluorescence signals from the 16th PCR cycle.

Abbreviations: Ct, cycle threshold; SARS-CoV-2, severe acute respiratory syndrome coronavirus-2;

**Table S4. Detailed clinical information for clinical specimens**

| **Patient characteristic** | **No. (%) positive results for:** | | | **No. (%) negative results for:** | | |
| --- | --- | --- | --- | --- | --- | --- |
|  | **SARS-CoV-2 (n=42)** | **Influenza A (n=40)** | **Influenza B (n=29)** | **SARS-CoV-2**  **(n=87)** | **Influenza A (n=71)** | **Influenza B (n=76)** |
| Gender | | | | | | |
| Male | 22 | 21 | 12 | 39 | 35 | 32 |
| Female | 20 | 19 | 17 | 48 | 36 | 44 |
| Age group (yrs) | | | | | | |
| ≤10 | 0 | 4 | 4 | 4 | 5 | 7 |
| 11-30 | 7 | 2 | 8 | 37 | 21 | 32 |
| 31-50 | 25 | 8 | 15 | 29 | 29 | 23 |
| 51-70 | 9 | 17 | 2 | 12 | 11 | 12 |
| 71-90 | 1 | 8 | 0 | 5 | 5 | 2 |
| ≥90 | 0 | 1 | 0 | 0 | 0 | 0 |
| Sample types | | | | | | |
| Nasopharyngeal swab | 36 | 28 | 25 | 66 | 45 | 53 |
| Oropharyngeal swab | 6 | 12 | 4 | 21 | 26 | 23 |
| Placement | | | | | | |
| Inpatient | 12 | 34 | 3 | 25 | 27 | 24 |
| Outpatient | 25 | 6 | 7 | 44 | 32 | 43 |
| Emergency department | 5 | 0 | 19 | 18 | 12 | 9 |

**Table S5. Results of intra-assay (repeatability) precision and inter-assay (reproducibility) precision.**

|  |  | **SARS-CoV-2 ORF1ab gene** | | | | **SARS-CoV-2 N gene** | | | | | **Influenza A M gene** | | | | | **Influenza B HA gene** | | | | | |
| --- | --- | --- | --- | --- | --- | --- | --- | --- | --- | --- | --- | --- | --- | --- | --- | --- | --- | --- | --- | --- | --- |
| **Concentration** | **Day** | **Intra -assay** | | **Inter-assay** | | **Intra -assay** | | **Inter-assay** | | | **Intra -assay** | | **Inter-assay** | | | **Intra -assay** | | | **Inter-assay** | | |
|  |  | **Mean±SD, ℃** | **CV (%)** | **Mean±SD, ℃** | **CV (%)** | **Mean±SD, ℃** | **CV (%)** | **Mean±SD, ℃** | **CV (%)** | **Mean±SD, ℃** | | **CV (%)** | **Mean±SD, ℃** | **CV (%)** | **Mean±SD, ℃** | | **CV (%)** | **Mean±SD, ℃** | | **CV (%)** |  |
| **500 copies/μL** | **1** | 64.49±0.28 | 0.43 | 64.64±0.33 | 0.51 | 55.69±0.19 | 0.34 | 55.75±0.19 | 0.34 | 45.93±0.24 | | 0.53 | 46.07±0.28 | 0.61 | 67.26±0.14 | | 0.21 | 67.56±0.25 | | 0.37 |  |
|  | **2** | 64.84±0.32 | 0.50 |  |  | 55.80±0.27 | 0.48 |  |  | 46.11±0.40 | | 0.87 |  |  | 67.72±0.28 | | 0.42 |  |  |  |  |
|  | **3** | 64.58±0.34 | 0.52 |  |  | 55.80±0.27 | 0.48 |  |  | 45.94±0.16 | | 0.35 |  |  | 67.60±0.17 | | 0.26 |  |  |  |  |
| **125 copies/μL** | **1** | 64.28±0.33 | 0.51 | 64.62±0.46 | 0.70 | 55.69±0.16 | 0.28 | 55.91±0.32 | 0.57 | 45.86±0.31 | | 0.68 | 45.86±0.29 | 0.63 | 67.45±0.30 | | 0.45 | 67.85±0.34 | | 0.51 |  |
|  | **2** | 65.04±0.26 | 0.40 |  |  | 55.79±0.38 | 0.72 |  |  | 45.68±0.08 | | 0.17 |  |  | 67.96±0.17 | | 0.26 |  |  |  |  |
|  | **3** | 64.67±0.25 | 0.38 |  |  | 55.71±0.18 | 0.32 |  |  | 45.80±0.28 | | 0.61 |  |  | 68.05±0.34 | | 0.50 |  |  |  |  |
| **31.3 copies/μL** | **1** | 63.87±0.36 | 0.57 | 64.47±0.52 | 0.81 | 55.84±0.18 | 0.32 | 55.81±0.37 | 0.66 | 45.47±0.38 | | 0.83 | 45.65±0.45 | 0.99 | 67.89±0.08 | | 0.12 | 68.02±0.38 | | 0.56 |  |
|  | **2** | 64.76±0.55 | 0.84 |  |  | 55.71±0.18 | 0.32 |  |  | 45.65±0.53 | | 1.16 |  |  | 68.27±0.57 | | 0.83 |  |  |  |  |
|  | **3** | 64.55±0.47 | 0.73 |  |  | 55.99±0.59 | 1.05 |  |  | 45.85±0.43 | | 0.93 |  |  | 67.99±0.17 | | 0.25 |  |  |  |  |

Intra- and inter-assay variability was calculated using MS2-VLPs samples representing high and low viral loads with all three viruses. Shown here are the results from three independent runs with each sample tested in quintuplicate.
Abbreviations: SARS-CoV-2, severe acute respiratory syndrome coronavirus-2; SD: Standard deviation; %CV: coefficient of variation.
